# A Pink Herring in the Colon: A Case Report of Eosinophilic Colitis Masking Invasive Adenocarcinoma of the Colon

**DOI:** 10.1155/2020/5641701

**Published:** 2020-03-31

**Authors:** David Malcolm Milne, Jason Rattan, Alyssa Muddeen, Amrit A. Rambhajan

**Affiliations:** General Surgery Department, General Hospital Port of Spain, Trinidad and Tobago

## Abstract

Eosinophilic colitis is an inflammatory condition in which the wall of the colon becomes infiltrated by eosinophils which stain densely pink on microscopy. It is an uncommon clinical entity which has a long list of differential diagnoses. We present a case of a patient whose biopsy at colonoscopy revealed eosinophilic colitis which led to a delay in the diagnosis and subsequent treatment of colon cancer. A 35-year-old male presented with a six-week history of right lower quadrant abdominal pain associated with diarrhoea and weight loss. Colonoscopy showed an inflamed cecum; computed tomography revealed a small ascitic fluid collection in the right iliac fossa. Biopsy showed eosinophilic colitis, and he was treated conservatively with albendazole and mesalamine. The patient failed to improve over the following month with continued weight loss. A repeat CT scan showed a right iliac fossa mass. A right hemicolectomy was performed with histopathology from the specimen showing mucinous adenocarcinoma. Eosinophilic colitis can mask colon cancer and should be considered a diagnosis of exclusion.

## 1. Introduction

The term red herring originates from William Cobbett who described the use of a smoked red herring to distract hunting dogs from their pursuit of a rabbit [1]. The phrase is now commonly used to describe information which may be misleading. Unfortunately, the practice of medicine is fraught with distracting information which may lead the physician to an incorrect diagnosis. Diagnostic errors can have dire consequences for both the patient and the doctor when it leads to a delay in treatment for mitotic processes such as colon cancer [2, 3].

Worldwide colon cancer is the third most common cancer, accounting for the 2nd greatest number of cancer related deaths [4]. It is therefore critical that doctors should be aware of potential pitfalls in the diagnosis and management of this common condition. Delays in diagnosis can lead to the disease being discovered at a more advanced stage worsening the patient's prognosis and leaving doctors at risk for litigation [2, 3].

The diagnostic algorithm for colon cancer incorporates history, examination, and imaging studies. Treatment is embarked upon after histological confirmation from a specimen obtained via endoscopy [5, 6]. Regrettably in some endoscopy cases may not be able to identify a cancer despite a biopsy of the lesion being taken [7–9].

Eosinophilic colitis is an inflammatory condition in which the wall of the colon becomes infiltrated by eosinophils which stain densely pink on microscopy. This case is that of a patient whose biopsy from colonoscopy revealed eosinophilic colitis which led to a delay in the diagnosis and treatment of colon cancer. Like a red herring, eosinophilic colitis was a distracting diagnosis which masked a more sinister pathology.

This report highlights potential pitfalls in diagnosing a patient with eosinophilic colitis.

## 2. Case Report

Mr. B.C. is a 35-year-old male patient who presented with a six-month history of right lower quadrant pain and weight loss of 20 pounds. He also complained of constipation with a decrease in bowel motion frequency from once per day to two per week.

On examination, B.C. was noted to be cachexic. Palpation of the abdomen revealed tenderness in the right iliac fossa; however, no signs of peritonitis were elicited. Complete blood count and renal and liver function tests were normal.

**Figure 1:**
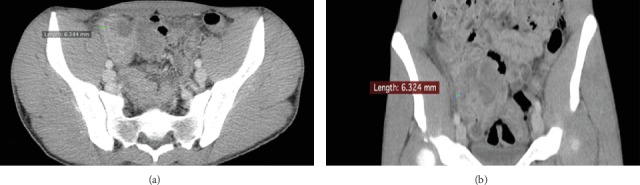
CT of the abdomen showing a thick-walled caecum, with adjacent fat stranding and a small volume of adjacent free fluid: (a) axial section and (b) coronal section.

Computed tomography (CT) of the abdomen and pelvis with intravenous contrast showed a thick-walled (6.3 mm) caecum with fat stranding and a small volume of adjacent free fluid (see Figure 1). These findings were reported as being consistent with inflammatory bowel disease.

**Figure 2:**
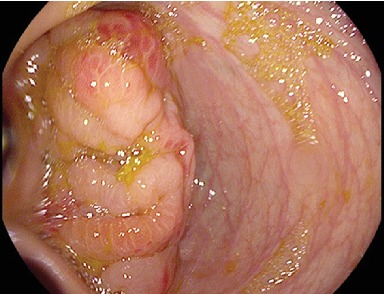
Caecal mass as seen on colonoscopy.

The patient subsequently had a colonoscopy which showed a mass at the caecal pole with edematous, inflamed tissue and pus (see Figure 2). Random biopsies were obtained from the inflamed colonic mucosa. Attempts at intubation of the ileocecal valve were unsuccessful. The rest of the colon appeared normal. Histology of the biopsy showed a marked increase in the number of chronic inflammatory cells dominated by tissue eosinophilia, consistent with a diagnosis of eosinophilic colitis. The specimen revealed no evidence of malignancy.

Considering the CT and histology findings, the patient's age, and the absence of a family history of malignancy, the findings at colonoscopy were interpreted as an inflammatory mass secondary to eosinophilic colitis. Stool microscopy showed no ova, cysts, and parasites. In conjunction with a gastroenterologist, the patient was started on an empiric course of albendazole and mesalamine.

**Figure 3:**
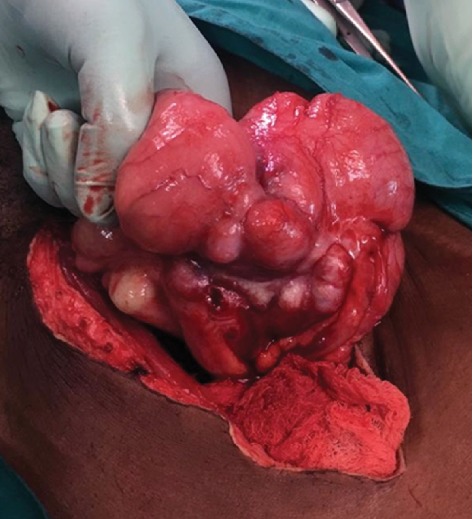
Caecal mass observed at laparotomy.

One month after his first presentation, Mr. B.C. represented reporting no improvement in symptoms, further weight loss of 10 pounds, and intractable abdominal pain. At this time, the patient was tender in the right lower quadrant of the abdomen with guarding. Repeat CT scan showed a fluid collection surrounding the caecum suggestive of an intra-abdominal abscess. Taking into account the new CT findings and continued clinical deterioration despite medical therapy, the decision was made to perform a laparotomy. Intraoperatively, a large caecal mass was found with an associated abscess and multiple enlarged mesenteric lymph nodes (see Figure 3). A right hemicolectomy was performed, and the patient had an uneventful recovery.

**Figure 4:**
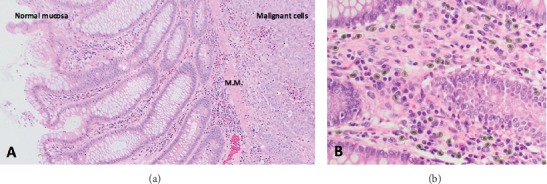
(a) Normal mucosa separated by an intact muscularis mucosa (M.M.) from malignant cells in the submucosa. (b) High power view of the mucosa overlying the tumour showing neutrophils outlined in fluorescent green; numbering >40 per h.p.f.

**Figure 5:**
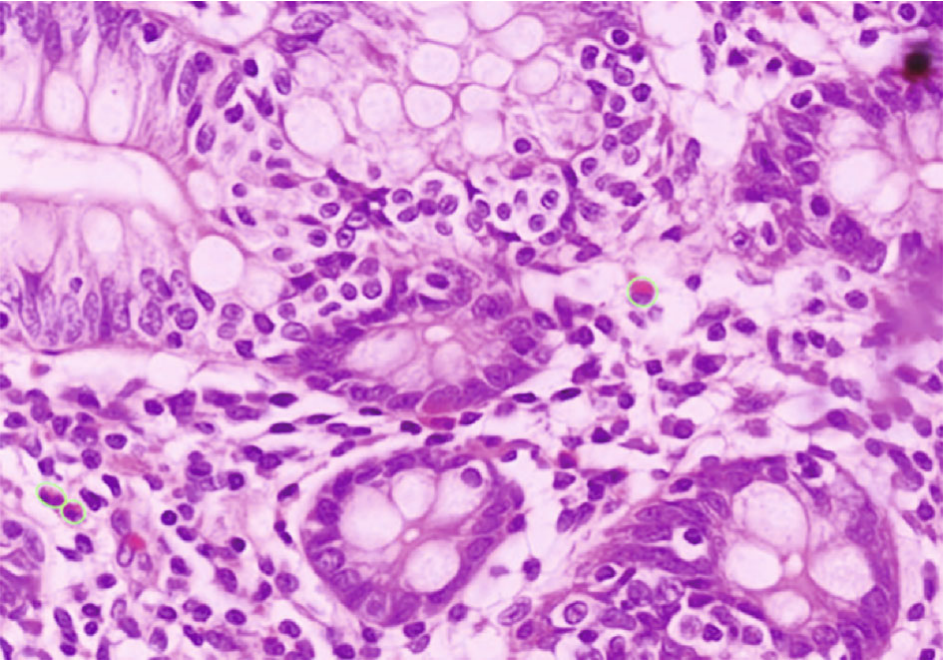
High power view of the mucosa of the ascending colon at a site distant from the tumour showing eosinophils outlined in fluorescent green; numbering <5 per h.p.f.

Histology of the surgical specimen revealed a moderately differentiated mucinous adenocarcinoma, pT3 pN1b. Of note, the tumour involved a relatively small area of the mucosa with the bulk of the tumour extending in the other layers of the bowel wall. The majority of the tumour was observed to be deep to mucosa, which showed no evidence of malignancy, and dense eosinophilic infiltrate of more than 40 per high powered field (Figure 4). This can be compared to Figure 5 which shows a section of the colonic mucosa taken from the ascending colon which showed less than 5 eosinophils per high powered field.

## 3. Discussion

Eosinophilic gastrointestinal disease (EGID) is a rare condition of unknown aetiology which is characterized by eosinophil-rich chronic inflammation of the gastrointestinal tract, in the absence of any known cause of eosinophilia. Initially described by Kaijser in 1937 [10], it can affect any segment of the bowel from the oesophagus to the rectum [11].

When EGID affects the colon, it is termed as eosinophilic colitis (EC). EC is the rarest form of EGID, with a prevalence of 2·3 per 100000 in adults [12]. Cases have been observed from infancy to adulthood with the peak incidence occurring in the 3rd to 5th decade of life [13].

The diagnosis of EC is made when a patient satisfies the following three criteria: (1) the presence of gastrointestinal symptoms, (2) histologically confirmed eosinophilic infiltration of the colon, and (3) other causes of colonic eosinophilia which have been excluded [13].

EC typically presents with vague abdominal complaints such as abdominal pain, nausea, vomiting, diarrhoea, constipation, and weight loss. Less frequently, patients may present with obstruction, perforation, intussusception, volvulus, and ascites [12].

Histological evaluation for EC is done via biopsy, typically obtained through endoscopy. There is no consensus in the literature regarding the level of eosinophils considered to be diagnostic; however, most authors used a threshold of 20 eosinophils per high powered field (h.p.f.) [14]. Using a single threshold appears to be inappropriate given that the normal values for tissue eosinophils vary along the length of the colon from <10 per h.p.f. in the rectum to >30 per h.p.f. in the caecum [14]. Furthermore, eosinophil number may vary widely among healthy individuals with varying exposure to infectious agents and allergens [15].

Few published cases of EC include quantification of eosinophilic infiltrates. Bates' review highlighted that out of 34 cases published in the English literature since 1959, just 7 cases included measurements of eosinophil density. Of these 7 cases, only 5 were genuinely idiopathic with the remaining cases due to a secondary cause. The author went on to question whether EC was an actual nosological entity and concluded that it should be regarded as a nonspecific reaction pattern [16]. With this in mind, a histological diagnosis of EC should trigger an aggressive search for an underlying source of eosinophila.

Numerous secondary causes of eosinophilic infiltration of the bowel wall have been identified in the literature. These include drugs (clozapine, rifampicin, and naproxen), parasitic infestation, scleredema, polymyositis, vasculitis, inflammatory bowel disease, radiotherapy, lymphomas, and carcinomas [17].

Eosinophils have a role in the host's reaction to tumours and are thought to have tumouricidal effects. In mice with tumour eosinophilia, there are decreased tumour progression and tumourigenicity. As it relates to colon cancer in humans, tumour eosinophilia is associated with an improved prognosis [18, 19]. Specifically, increasing tumour eosinophil counts are significantly associated with higher degrees of tumour differentiation, lower T and N staging, an absence of vascular invasion, and improved cancer-specific survival [20].

Colon cancers can contain large numbers of eosinophils, and they are often observed in tumour specimens. Harbaum et al.'s histological review of colon cancer specimens revealed intratumoural eosinophils in 86% of specimens and peritumoural eosinophils in 75% of specimens [20]. Given the frequency with which eosinophils are observed in colon cancer, extreme caution should be employed before a clinician accepts a diagnosis of EC in a patient who has other features suggestive of carcinoma.

This case highlights the importance of a multidisciplinary approach to the management of patients. With improved communication between surgery, gastroenterology, and pathology, perhaps, this patient may have had a repeat biopsy following his colonoscopy. This could have led to an earlier diagnosis of colon cancer and avoided a delay in surgery. Earlier surgery would have afforded the surgeons the opportunity to operate on a patient with better nutritional status and thus reduced the risk of postoperative complications [21]. More importantly, delays in surgery for colon cancer result in a higher chance of advanced disease at the time of surgery and a decreased probability of survival [22].

As a consequence of the nonspecific nature of the presentation of EC, there have been many reports of it being confused preoperatively with other diagnoses including inflammatory bowel disease, acute appendicitis, and small bowel obstruction [23]. It is, however, surprisingly uncommon for a preoperative diagnosis of eosinophilic colitis to be incorrect as determined by surgical pathology. To the best of our knowledge, this case is one of only 2 cases in the literature where preoperative colonoscopy biopsies showed evidence of eosinophilic colitis and the patient went on to have surgical pathology specimens revealing colon cancer [19].

## 4. Conclusion

Eosinophilic colitis is an uncommon clinical condition whose diagnostic criteria are not elucidated in the literature, and it may not be a true nosological entity. Increased numbers of eosinophils can be observed in the colon in a number of disease conditions, including colon cancer. Given the potential for adverse outcomes because of a misdiagnosis, an extensive search for secondary causes of eosinophilia should be undertaken employing a multidisciplinary approach before a patient is diagnosed with eosinophilic colitis.
